# Outer Membrane Protein A (OmpA): A New Player in *Shigella flexneri* Protrusion Formation and Inter-Cellular Spreading

**DOI:** 10.1371/journal.pone.0049625

**Published:** 2012-11-14

**Authors:** Cecilia Ambrosi, Monica Pompili, Daniela Scribano, Carlo Zagaglia, Sandro Ripa, Mauro Nicoletti

**Affiliations:** 1 Dip. di Scienze Sperimentali e Cliniche, Università “G. D’Annunzio’ di Chieti, Chieti, Italy; 2 Dip. di Sanità Pubblica e Malattie Infettive Università “Sapienza” di Roma, Rome, Italy; 3 Dip. di Biologia, Università “Roma TRE”, Rome, Italy; 4 Dip. di Biologia Molecolare, Cellulare e Animale Università di Camerino, Camerino (MC), Italy; Universidad de Costa Rica, Costa Rica

## Abstract

Outer membrane protein A (OmpA) is a multifaceted predominant outer membrane protein of *Escherichia coli* and other *Enterobacteriaceae* whose role in the pathogenesis of various bacterial infections has recently been recognized. Here, the role of OmpA on the virulence of *Shigella flexneri* has been investigated. An *ompA* mutant of wild-type *S. flexneri* 5a strain M90T was constructed (strain HND92) and it was shown to be severely impaired in cell-to-cell spreading since it failed to plaque on HeLa cell monolayers. The lack of OmpA significantly reduced the levels of IcsA while the levels of cell associated and released IcsP-cleaved 95 kDa amino-terminal portion of the mature protein were similar. Nevertheless, the *ompA* mutant displayed IcsA exposed across the entire bacterial surface. Surprisingly, the *ompA* mutant produced proper F-actin comet tails, indicating that the aberrant IcsA exposition at bacterial lateral surface did not affect proper activation of actin-nucleating proteins, suggesting that the absence of OmpA likely unmasks mature or cell associated IcsA at bacterial lateral surface. Moreover, the *ompA* mutant was able to invade and to multiply within HeLa cell monolayers, although internalized bacteria were found to be entrapped within the host cell cytoplasm. We found that the *ompA* mutant produced significantly less protrusions than the wild-type strain, indicating that this defect could be responsible of its inability to plaque. Although we could not definitely rule out that the *ompA* mutation might exert pleiotropic effects on other *S. flexneri* genes, complementation of the *ompA* mutation with a recombinant plasmid carrying the *S. flexneri ompA* gene clearly indicated that a functional OmpA protein is required and sufficient for proper IcsA exposition, plaque and protrusion formation. Moreover, an independent *ompA* mutant was generated. Since we found that both mutants displayed identical virulence profile, these results further supported the findings presented in this study.

## Introduction

*Shigella flexneri* is a Gram-negative facultative intracellular pathogen that causes bacillary dysentery, a major public-health problem principally in developing countries. Most of the genes required for invasion of epithelial cells and cell-to-cell spread are located on the large virulence plasmid. Important aspects of *Shigella* pathogenesis are the ability to penetrate and multiply within colonic epithelial cells [1], [2]. Invasion of epithelial cells proceeds by bacteria-induced phagocytosis, rapid lysis of the phagocytic vacuole, multiplication inside the cytoplasm of host cells, and subsequently intra- and inter-cellular spreading of the infection within epithelial tissues by polymerizing cell actin and forming long F-actin comet tails which propel bacteria through the cell cytoplasm and to the cell periphery [2], [3], [4], [5], [6]. *Shigella* cell-to-cell spread requires the expression and polar surface exposition of IcsA (VirG), a 110-kDa autotransporter protein encoded on the *Shigella* large virulence plasmid. Once translocated across the outer membrane (OM), IcsA exposes its N-terminal α-domain (the passenger domain) on the bacterial surface interacting with the eukaryotic proteins vinculin and neural Wiskott-Aldrich syndrome protein (N-WASP). N-WASP then recruits the host Arp2/3 complex, which induces polymerization of host globular actin into filamentous actin and cross-links these new filaments at 70° angles [7], [8], [9], [10], [11], [12]. In exponentially-growing bacteria IcsA is found to be exclusively exposed at the old bacterial pole [13], [14]. Although the mechanism driving the polar localization of IcsA is still unclear, several experimental evidences indicate that IcsA inserts directly at the pole [13], [15], [16]. Furthermore, *Shigella* moves to adjacent cells via protrusion formation without leaving the intracellular compartment. Protrusions are membrane-bound cell extensions that are driven by the bacterium and that propel it into adjacent cells. Protrusions, which may extend tens of microns from the cellular surface, are characterized by the presence of a bacterium at its tip [6]. By a process which likely resembles macropinocytosis, contact with the membrane of an adjacent cell is followed by the uptake of the bacterium [17], leading to the spreading of the infection to neighboring epithelial cells. Several host cell proteins have been implicated in protrusion-mediated *Shigella* cell-to-cell spread, suggesting that a distinct set of actin regulatory factors interacts with motile bacteria after they contact the plasma membrane [18]. Although actin polymerization and assembly are required for protrusion formation, the specific molecular mechanisms responsible for this phenomenon are poorly defined. Furthermore, recent experimental evidences indicate that actin nucleation processes, involved in protrusion formation, may be independent of the activity of the Arp2/3 complex [12]. In this context, it has been recently reported that protrusion formation and inter-cellular spreading depend on actin polymerization that requires the activation of the Diaphanous formin Dia [12]. Formins are a family of ubiquitous expressed proteins that, in contrast to the Arp2/3 complex, initiate *de novo* actin polymerization leading to cross-linking of actin polymers in parallel arrays [12].

OmpA is a 35 kDa monomeric protein embedded in the bacterial OM as a β-barrel protein, highly conserved among Gram-negative bacteria [19]. OmpA is thought to play a pivotal role, along with other bacterial components, in the structural integrity of the OM and it has been reported to be an important virulence factor of several human pathogens [19], [20]. Recent studies have suggested that OmpA, likely because of its capacity to interact with a variety of host tissues, has to be considered a pleiotropic molecule which plays different roles in the mechanism of virulence of different bacterial pathogens. Among other important features, it has been reported that OmpA may serve as an adhesin/invasin as well as an immune evasin, a participant in biofilm formation, a receptor for several bacteriophages and as a mediator of F-dependent conjugation [19], [20], [21]. Furthermore, in an experimental animal model, OmpA of *S. flexneri* 2a has been identified as a novel molecule coordinating the innate and adaptive immune responses, strongly indicating that OmpA may also represent a promising antigen in vaccine development [22], [23]. In spite of its importance in the immune response of *S. flexneri*, to date no reports have documented the role of OmpA on the virulence of *S. flexneri*.

In this study we have investigated the role of OmpA in the mechanism of virulence of *S. flexneri*. Our results indicate that OmpA is absolutely required for proper IcsA presentation, plaque and protrusion formation.

## Materials and Methods

### Bacterial Strains, Plasmids, Cell Lines, and Growth Conditions

Bacterial strains and plasmids used in this study, and their relevant characteristics, are listed in Table 1. *S. flexneri* strains were routinely plated on tryptic soy broth agar plates containing 0.01% Congo red (CR). *Escherichia coli* and *S. flexneri* strains were grown in Luria Bertani broth (LB; Difco). When necessary, antibiotics were used at the following concentrations: chloramphenicol (Cm), 30 µg ml^−1^, kanamycin (Km), 25 µg ml^−1^. HeLa cells from ATCC were grown in Dulbecco’s modified Eagle’s medium supplemented with 10% fetal bovine serum and grown at 37°C in the presence of 5% CO_2_.

**Table 1:** Strains, plasmids, and primers used in this study.

Strain or plasmid or primer	Description	Source or reference
**Strains**		
**M90T**	Wild-type *S. flexneri* serotype 5a	[61]
**HND92**	M90T Δ*ompA*; Km^r^	This study
**HND99**	M90T Δ*ompA*; susceptible	This study
**SC560**	M90T Δ*icsA*::Ω; Sm^r^	[62]
**JW0940**	BW25113 Δ*ompA*; Km^r^	Keio Collection
**DH10b**	*E. coli* K-12	Gibco BRL
**Plasmids**		
**pACYC184**	Cloning vector; Cm^r^, Tc^r^	Fermentas
**pOA**	pACYC184 carrying *ompA*; Cm^r^	This study
**pKD4**	Template plasmid carrying a Km^r^ gene with FLP recognition target sequence	[52]
**pCP20**	FLP helper plasmid; pSC101 replicon (Ts) *bla cat* Flp (λR*p*) *cI857* Ap^r^ Cm^r^	[52]
**Primers** a		
**ompA2FW**	5′-CGCGGGATCCAGGCTTGTCTGAAGCGGTTT-3′	This study
**ompA2RV**	5′GCGGAAGCTTGGCATTGCTGGGTAAGGAAT-3′	This study
**ompADFW**	5′ATGAAAAAGACAGCTATCGCGATTGCAGTGGCACTGGCTGTGTAGGCTGGAGCTGCTTC-3′	This study
**ompADRV**	5′AACGTCTTTGATACCTTTAACTTCGATCTCTACGCGACGCATATGAATATCCTCCTTAG-3′	This study

a Restriction sites are underlined.

### Construction of the *ompA* Mutant Strain HND92

The Δ*ompA* mutation carried by the *E. coli* K-12 strain JW0940 was P1 transduced into the *S. flexneri* 5a wild-type strain M90T (Table 1). Transductants were selected on CR plates supplemented with kanamycin and their structure was verified by PCR analysis (using nearby *ompA* specific primers; Table 1) and sequencing. The absence of the OmpA protein was verified by Western blot. One transductant, named HND92, was chosen for further studies.

### DNA Manipulations

DNA extraction, isolation of plasmids, restriction digestion, electrophoresis, purification of DNA fragments, and electroporation were performed by standard methods [24]. Real Time PCR analysis of *icsA* expression was performed as previously described [25]. The entire *ompA* gene, including its promoter region, was amplified by PCR, using the primer pair ompA2FW and ompA2RV (Table 1), designed on available genome sequence of strain M90T (GenBank CM001474.1). The PCR product was *Bam*HI/*Hind*III digested and cloned into the corresponding sites of the pACYC184 vector (Fermentas), generating plasmid pOA. The correct insertion of the *ompA* gene in pACYC184 was verified by PCR and sequencing prior to be introduced by electroporation into strains HND92 and HND99 (Table 1) for complementation experiments. OmpA expression was verified by Western blot analysis.

### Whole Cell Extracts, Secreted Proteins and Cell Fractionation

Whole cell extracts (WCEs) were prepared by lysing concentrated exponentially-growing bacteria in 1×Laemmli buffer. WCEs of infected HeLa cells were prepared by directly lysing washed infected monolayers with 1×Laemmli buffer. Type III secretion (T3S) system secreted proteins were prepared from bacteria grown to mid-log phase as previously described [26]. Briefly, bacteria were washed and re-suspended in 1×PBS, CR was added to a final concentration of 7 µg ml^−1^, and bacteria were incubated for 30 min. After incubation, the bacteria were pelleted by centrifugation and the supernatant fraction was filtered through a 0.22 µm-pore filter. Secreted proteins were concentrated by trichloroacetic acid (TCA) treatment. TCA-pellets were re-suspended in 50 µl of 1×Laemmli buffer and stored at −20°C. Crude membrane and cytosolic/periplasmic bacterial fractions were prepared from equal numbers of exponentially-growing bacteria. Bacterial pellets were re-suspended in sonication buffer (150 mM NaH_2_PO_4_, 300 mM NaCl, 1 mM PMSF, and 1 mg ml^−1^ lysozyme), and incubated for 30 min on ice. At the end of the incubation period, cells were disrupted by sonication (Soniprep MSE 150, Sanyo). After removal of cell debris, the cleared-lysates were centrifuged at 18,000×*g* for 30 min at 4°C, and pellets, corresponding to the crude membrane fraction, were re-suspended in 1×Laemmli buffer, while supernatants, corresponding to the cytosolic and periplasmic fractions, were concentrated by TCA precipitation. TCA-precipitated proteins were washed with 90% acetone and re-suspended in 1×Laemmli buffer. When indicated, fractions were split and incubated at 37°C or at 100°C for 10 min.

### Sodium Dodecylsulphate-polyacrylamide Gel Electrophoresis (SDS-PAGE) and Western Blot

Proteins were resolved by 12.5% Tris-glycine SDS-PAGE [27], and transferred onto PVDF membranes (Hybond-P GE-Heathcare Bio-Sciences). Western blot analysis was performed using the following antibodies: rabbit polyclonal anti-OmpA and anti-IcsA, mouse monoclonal anti-IpaB and anti-IpaC antibodies (gifts from N.V. Prasadarao, M.L. Bernardini and A. Phalipon, respectively); polyclonal anti-host cytosolic phospholipase A2 (cPLA2α) and anti-phospho-cPLA2α antibodies were from Cell Signal Technologies (Danvers, MA) and Santa Cruz Biotechnology (Santa Cruz, CA), respectively. Anti-mouse and anti-rabbit conjugated to horseradish peroxidase (Bio-Rad) were used as secondary antibodies. Blots were visualized by enhanced chemiluminescence system (GE-Healthcare Bio-Sciences).

### Preparation of LPS and Serum Sensitivity Assay

LPS were prepared from overnight bacterial cultures essentially as described by Hitchcock and Brown [28]. Aliquots of LPS preparations were incubated at 100°C for 5 min prior to loading on 13% Tris-glycine SDS-PAGE, and silver-stained as described [29]. For serum sensitivity assays, the wild-type strain, the *ompA* mutant strain HND92, and the *E. coli* K-12 strain DH10b were grown to mid-log phase, collected by centrifugation, washed and diluted in PBS (pH 7.4). *S. flexneri* (5×10^5^ CFU ml^−1^) and *E. coli* (5×10^6^ CFU ml^−1^) strains were suspended in the presence of 10% normal human serum (NHS, Lonza), and incubated at 37°C for 2 hrs. The number of CFUs was determined at 30 min intervals by plating serial dilutions onto CR plates. Serum heated at 56°C for 30 min (heat-inactivated normal human serum; HIS) was used as control in all experiments. Percent survival was calculated as the average of CFUs that survived exposure to NHS divided by the CFUs that survived exposure to HIS at each time point×100.

### Invasiveness, Plaque Assay, Actin Tails and Protrusion Formation

Invasion of non-confluent HeLa cell monolayers was carried out using the gentamicin protection assay at a multiplicity of infection (MOI) of 20, as previously described [30]. The percentage of cell invasion (percent of HeLa cells containing three or more intracellular bacteria) and the formation of protrusions (defined as extensions of the plasma membrane outside the normal contour of the HeLa cell that extended more than a bacterial length and that contained a bacterium at its tip) were evaluated with Giemsa-stained infected monolayers. The frequency of membrane protrusions was quantified 1 h post-infection by enumerating membrane protrusions divided by the number of infected HeLa cells. A minimum of 300 infected HeLa cells were considered for each experiment. Multiplication of bacteria within HeLa cells was assayed essentially as previously described [4]. Non-confluent HeLa cells were infected (MOI = 100) centrifuged for 20 min at 37°C. Washed monolayers were covered with 2 ml of minimal essential medium containing gentamicin (50 µg ml^−1^) (time zero). Incubation was usually carried out for 5 h and two plates were removed 1, 3 and 5 hours. One plate was washed and Giemsa stained to calculate the percentage of infected cells at each time point. The other plate was washed, cells were trypsinized, counted and then lysed with 0.5% of sodium deoxycholate in distilled water. Dilutions of bacterial suspensions were plated onto CR plates. The average number of bacteria per infected cell was calculated. Experiments were repeated at least three times for each strain tested. Results represent the means of three experiments. Plaque assays were performed on confluent HeLa cell monolayers, as previously described [31], using a MOI of 4. After 72 h, cells were fixed and plaques were visualized by Giemsa-staining. To detect F-actin comet tails, infected HeLa cells were fixed for 10 min in 3.7% (w/v) paraformaldehyde, permeabilised with 0.2% Triton X-100 for 10 min and actin tails visualized by staining with rhodamine-conjugated phalloidin (Sigma). Bacterial and cellular DNAs were labelled with 0.2 μg ml^−1^ 4′,6′-diamidino-2-phenylindole (DAPI, Molecular Probes). The frequency of actin tails was calculated at 2 h post-infection, in samples of HeLa cells carrying up 8 to 10 internalized bacteria, as the number of bacteria displaying apparently normal F-actin comet tails divided by the total number of intracellular bacteria. A minimum of 100 infected HeLa cells were considered for each experiments. Experiments were repeated at least three times. Images were recorded with a Leica DM5000B microscope equipped with DFX340/DFX300 camera and processed using the Leica Application Suite 2.7.0.R1 software (Leica).

### Quantitative Detection of IcsA

Quantification of the relative amounts of IcsA on intact bacteria was achieved essentially as previously described [32] as well as by immuno-dot blot analysis. Briefly, equivalent volumes of exponentially-growing bacteria [normalized to the optical density at 600 nm (OD_600_)] were washed in PBS and fixed for 15 min in 3.7% (w/v) paraformaldehyde. Washed bacteria were then re-suspended in a 1∶100 dilution of *S. flexneri* type 5 antiserum (Denka Seiken) or in a 1∶100 dilution of rabbit anti-IcsA antiserum and incubated for 1 h. After washing, the bacterial suspensions treated with the anti-IcsA antiserum were suspended in a 1∶100 dilution of HRP-conjugated goat anti-rabbit secondary antibody (Bio-Rad) and incubated for 1 h. After washing, bacteria were re-suspended in 100 µl of peroxidase substrate (Rockland). After 10 min of incubation, the supernatants of each bacterial suspension were transferred to a 96-well flat-bottom plate and absorbance was measured at 370 nm using a microplate reader equipped with Gen5 data analysis software (BioTek Instruments). Immuno-dot blot experiments were also carried out to quantify IcsA on intact bacteria [33]. Briefly, exponentially-growing bacteria were pelleted by centrifugation and re-suspended to equivalent volumes in PBS and 5 µl of each bacterial suspension was spotted onto a nitrocellulose membrane (Whatman) and allowed to air dry. Membranes were then processed using standard Western blotting procedures using diluted (1∶20.000) rabbit polyclonal anti-IcsA antibody. Densitometry was performed using ImageJ software [34].

### Assessment of Membrane Integrity

To assess membrane integrity, the red- and green-fluorescent dyes propidium iodide (PI) and SYTO9 from the LIVE/DEAD BacLight kit (Molecular Probes, Invitrogen) were used in combination, following manufacturer’s instructions. Exponentially-growing bacteria (about 5×10^8^ bacteria ml^−1^) were washed twice with 0.85% NaCl and re-suspended in 1 ml of 0.85% NaCl containing equal volumes (3 µl each) of PI (20 mM in DMSO) and SYTO 9 (3.34 mM in DMSO). The mixture was incubated at 25°C in the dark for 15 min. Samples of stained cells (200 µl each) were dispensed into separate wells of a 96-well flat-bottom microplate and fluorescence determined with a microplate reader equipped with Gen5 data analysis software (BioTek Instruments). Excitation wavelength was centered at 485±20 for both dyes, while emission wavelengths were centered at 528±20 and 645±40 for SYTO9 and PI, respectively. The intensities of the green (SYTO9) and red (PI) emission of each suspension were acquired as fluorescence intensity units and the green/red ratios were calculated. Isopropanol-treated cells were used as controls. Three independent experiments were performed, each in duplicate.

### Statistical Analysis

The statistical significance of data was determined by the Student’s t-test. A *P* value of <0.05 was considered to be statistically significant.

## Results

### Construction of the *ompA* Mutant Strain HND92

**Figure 1:**
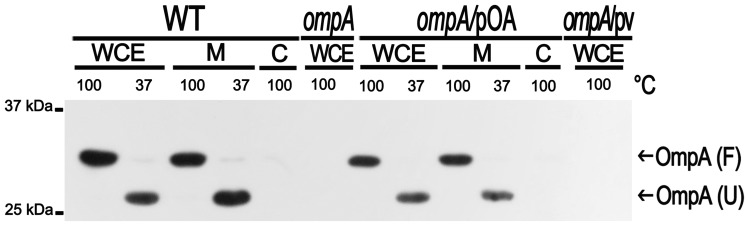
Western blot analysis of OmpA expression. OmpA expression by the wild-type *S. flexneri* 5a strain M90T (WT), the *ompA* mutant (*ompA*), the *ompA* mutant complemented with plasmid pOA (*ompA*/pOA) and the *ompA* mutant complemented with the empty vector pACYC184 (*ompA*/pv) is shown.WCE, whole cell extract; M, crude membrane fraction; C, cytosolic and periplasmic fraction. Fractions were suspended in 1×Laemmli buffer and heated at 37°C or at 100°C for 10 min before SDS-PAGE to evaluate the unfolded (U) and folded (F) forms of OmpA [38], [39]. PVDF filters were probed using an anti-OmpA polyclonal antiserum. The positions of U and F forms of OmpA are indicated by arrows at the right side. Size markers (kDa) are shown at the left side.

To investigate the role of OmpA in *S. flexneri* virulence, a Δ*ompA* mutation carried by the *E. coli* K-12 strain JW0940 (Table 1) was introduced into the wild-type *S. flexneri* strain M90T by P1 transduction. The correct structure of tranductants was verified (see Material and Methods for details) and one of them, named HND92, was used in all experiments. HND92 was able to bind Congo red and did not present growth defects since, in LB medium, there was no difference between the optical density of HND92 and that of the wild-type strain (data not shown). Exploiting the known cross-reactivity between enterobacterial species [35], [36], [37], we used polyclonal rabbit anti-OmpA antibody, raised against the *E. coli* OmpA protein, to detect OmpA in *S. flexneri*. A band of the expected size of OmpA (35 kDa) was detected by Western blot of WCE of the wild-type strain while no detectable band corresponding to OmpA was observed in WCE of HND92 (Fig. 1). Complementation of the *ompA* mutation with plasmid pOA, which carries the *ompA* gene and its promoter region cloned into the plasmid vector pACYC184 (Table 1), restored OmpA expression (Fig. 1). Moreover, proper localization within the membrane fraction and folding of OmpA was also assayed. Native OmpA of *E. coli* is known to present typical heat-modifiable mobility of β-barrels in SDS-PAGE which are known to form a compact SDS-resistant structure at low-temperature (37°C) that unfolds upon heating at higher temperature (100°C) [38], [39]. Indeed, both OmpA of *S. flexneri* and OmpA expressed by recombinant plasmid pOA behaved as native *E. coli* OmpA, since both proteins were found to be strictly localized within the membrane fraction and presented the expected heat-variable mobility (Fig. 1 and data not shown). These results indicated that OmpA of *S. flexneri* presented the same relevant biological characteristics of OmpA of *E. coli*.

### The *ompA* Mutation does not Affect IpaABCD Secretion

**Figure 2:**
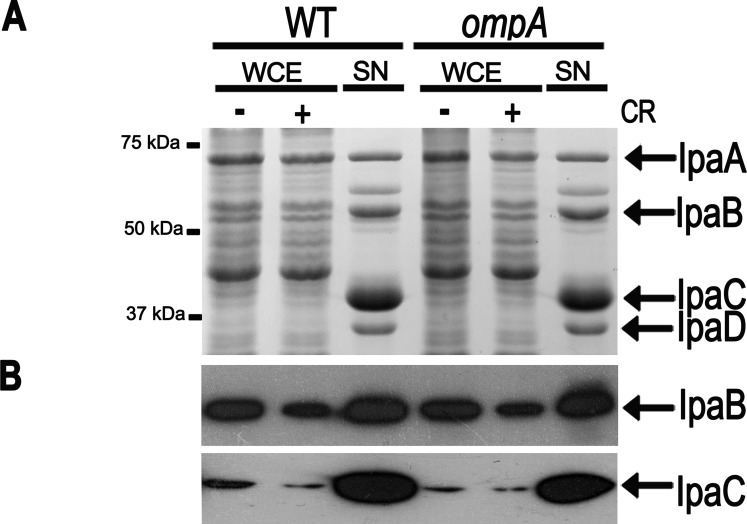
The *ompA* mutation did not affect either production or secretion of IpaABCD proteins. Proteins present in WCEs and concentrated culture supernatants (SN) of the wild-type strain (WT) and of the *ompA* mutant (*ompA*), following exposure of bacteria to 7 µg ml^−1^ of Congo Red (CR), were stained with Comassie brilliant blue (A) or transferred to a PVDF membrane and probed using anti-IpaB and anti-IpaC antibodies (B). The positions and names of the various proteins are indicated at the right side. Size markers (kDa) are shown at the left side.

To assess whether the *ompA* mutation affected the ability of *S. flexneri* to synthesize and secrete IpaABCD effector proteins, we compared IpaABCD synthesis and secretion in the wild-type and in the *ompA* mutant. Exponentially-growing bacteria were incubated in the presence of CR in order to activate the T3S system [26], [40]. SDS-PAGE analysis of Comassie brilliant blue staining of whole bacterial extracts and of concentrated culture supernatants indicated that neither the production nor the secretion of IpaABCD proteins were affected by the inactivation of the *ompA* gene (Fig. 2A). Accordingly, immunoblotting using anti-IpaB and anti-IpaC antibodies showed no difference in the expression and secretion of both proteins (Fig. 2B). These results indicated that the *ompA* mutant has a functional T3S system.

### The *ompA* Mutant Presents an Altered Invasive Phenotype

**Figure 3:**
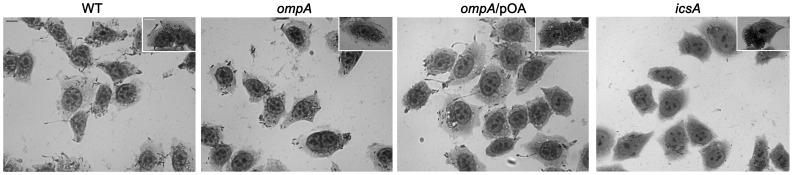
The *ompA* mutant retained wild-type invasion efficiency. Semi-confluent HeLa cell monolayers were infected with the wild-type strain (WT), the *ompA* mutant (*ompA*), and the *ompA* mutant complemented with plasmid pOA (*ompA*/pOA), at a MOI of 20. Monolayers were fixed with methanol and Giemsa-stained for microscopic evaluation at 1 h and 3 h post-infection (insets). The *icsA* mutant SC560 strain was included as control (*icsA*). Representative images of at least four independent experiments are shown. Scale bar, 10 µm.

**Figure 4:**
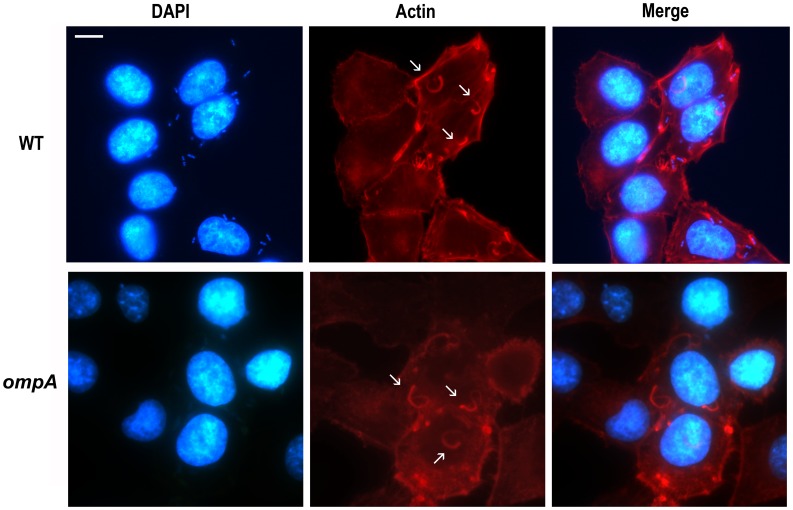
The *ompA* mutant retained the ability to form wild-type F-actin comet tails. Semi-confluent HeLa cell monolayers were infected with the wild-type strain (WT), the *ompA* mutant (*ompA*), and the *ompA* mutant complemented with plasmid pOA (*ompA*/pOA). Actin was stained with rhodamine-conjugated phalloidin (red). Nuclei and bacterial DNA were stained with DAPI (blue). Representative images of double immunofluorescent microscopy are shown. Images are representatives of at least three independent experiments. Images were captured using a Leica camera and processed using Qwin software (Leica). Arrows point representative bacteria exhibiting normal F-actin comet tail formation. Scale bar, 10 µm.

**Figure 5:**
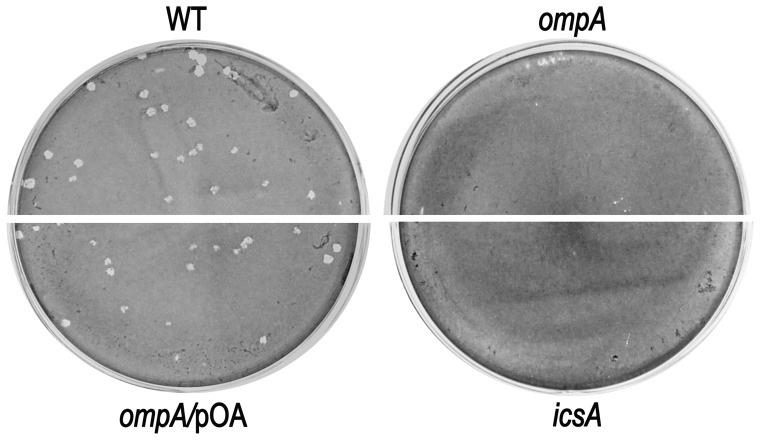
The *S. flexneri ompA* mutant failed to plaque on HeLa cell monolayers. Confluent HeLa cell monolayers were infected with the wild-type strain (WT), the *ompA* mutant (*ompA*), and the *ompA* mutant complemented with plasmid pOA (*ompA*/pOA), at a MOI of 4. The *icsA* mutant SC560 strain (*icsA*) was included as a plaque negative control. Cells were Giemsa-stained and photographed 72 h post-infection. Images are representative of at least three independent experiments.

The ability of the *ompA* mutant to invade and to multiply within semi-confluent HeLa cell monolayers was compared to that of wild-type, The *ompA* mutant was proficient both in host cell invasion and intracellular multiplication (Fig. 3). Interestingly, at 5 h post-infection HND92 displayed a reduced invasion phenotype. On average, we found that the number of HND92 bacteria in the cytoplasm was higher than that of wild-type (216±22 and 158±18 bacteria per infected HeLa cell, respectively). In contrast, whereas the percentage of cells infected by the wild-type strain was 82.1±12.0%, the proportion of cells infected by HND92 was 46.2±6.3% (*P*≤. 0.05). Moreover, at 3 h post-infection striking differences regarding the intracellular bacterial distribution were observed in Giemsa stained monolayers (Fig. 3). While the wild-type strain displayed the characteristic random distribution within the cytoplasm of Hela cells, the *ompA* mutant showed a less randomly distributed behavior with bacteria organized in parallel arrays and which seemed to be entrapped within the cell cytoplasm. This intra-cellular distribution was reminiscent of that of *mlaA*/*vacJ S. flexneri* mutants impaired in inter-cellular spread [41], and significantly different to that shown by the *icsA* mutant strain SC560 (Table 1) which displayed bacteria growing in cluster in the cell cytoplasm (Fig. 3, insets). Moreover, random distribution of bacteria within the cytoplasm of HeLa cells was restored when the *ompA* mutant was complemented with plasmid pOA, indicating that OmpA is required for normal distribution of *S. flexneri* in the cell cytoplasm. Noteworthy, differently from wild-type, protrusions were noticed only sporadically in HeLa cells infected with the *ompA* mutant. This finding prompted us to measure the frequency of protrusion formation. Assuming a relative arbitrary value of 100% for the number of protrusions (per infected HeLa cells) formed by the wild-type strain 1 h post-infection, under these conditions the *ompA* mutant scored about 6.9±2.4%, while HND92/pOA displayed about 89.7±6.9% (*P*≤0,01). These results clearly indicated that the lack of OmpA was responsible of the protrusion defect of the *ompA* mutant. Cytoplasmic entrapment of bacteria and the dramatic decrease in protrusion formation were highly suggestive of impaired actin-based motility (ABM). Therefore, the ability of the *ompA* mutant to form F-actin comet tails and to plaque on HeLa cells was determined. As for the wild-type, the *ompA* mutant was found to be proficient in the formation of apparently normal F-actin comet tails (Fig. 4), as determined by measuring the frequency of bacteria presenting apparently normal actin tails per infected HeLa cell (22.2±1.5% and 24.2±2.7% for wild-type strain and the *ompA* mutant, respectively; *P*≥0.5). Unexpectedly, despite the formation of proper actin tails, the *ompA* mutant failed to plaque on confluent HeLa cell monolayers (Fig. 5), even when HeLa cells were infected at higher MOIs or extending the period of incubation (data not shown). The ability to produce plaques at almost wild-type level was rescued by complementing the *ompA* mutant with plasmid pOA (0.9–1.2 mm against 1.0–1.2 mm of wild-type, after 72 h of incubation) (Fig. 5), indicating that a functional OmpA protein is required and sufficient for the inter-cellular spread of *S. flexneri*.

**Figure 6:**
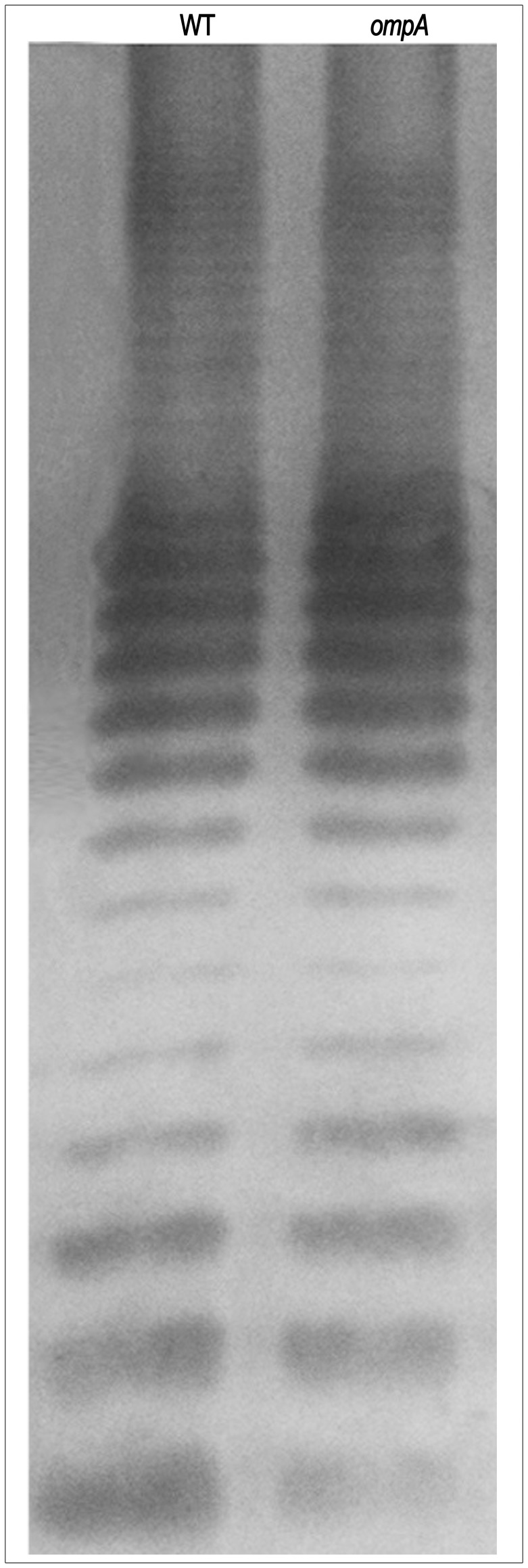
LPS profiles of the wild-type strain (WT) and of the *ompA* mutant (*ompA*). LPS was extracted as described in Materials and Methods, resolved by SDS-13%PAGE and silver stained.

Previous studies have reported that mutations of genes involved in the biosynthesis of the LPS of *S. flexneri* lead to the inability to plaque [42], [43]. To determine whether the lack of OmpA could have altered LPS biosynthesis, thus causing the inability of the *ompA* mutant to plaque, we first evaluated the sensitivity of the *ompA* mutant to the killing of normal human serum (NHS), since it has been reported that wild-type LPS of *S. flexneri* is required to confer resistance to NHS killing [44]. To this end, mid-log cultures of the *ompA* mutant, of the wild-type strain and of the *E. coli* K-12 DH10b (used as a serum-sensitive control) were incubated for 2 h (30 min intervals) in the presence of 10% of NHS or with heat-inactivated normal human serum (HIS). The percent survival to killing of the *ompA* mutant was indistinguishable to that of the wild-type strain while, as expected, the *E. coli* DH10b strain was highly sensitive to NHS. There was a greater than 5 log difference between the CFUs of *E. coli* DH10b rescued after 30 min of incubation in 10% NHS and the CFUs of wild-type and the *ompA* mutant after 2 h of incubation (data not shown). Next, LPS preparations were prepared as described in Materials and Methods. Aliquots were subjected to SDS-13%-PAGE and visualized by silver staining. Figure 6 shows that the *ompA* mutant produced the typical LPS structure seen in the wild-type strain, thus definitively ruling out that the lack of OmpA might have altered LPS biosynthesis and that the inability to plaque of the *ompA* mutant was due to LPS alterations.

**Figure 7:**
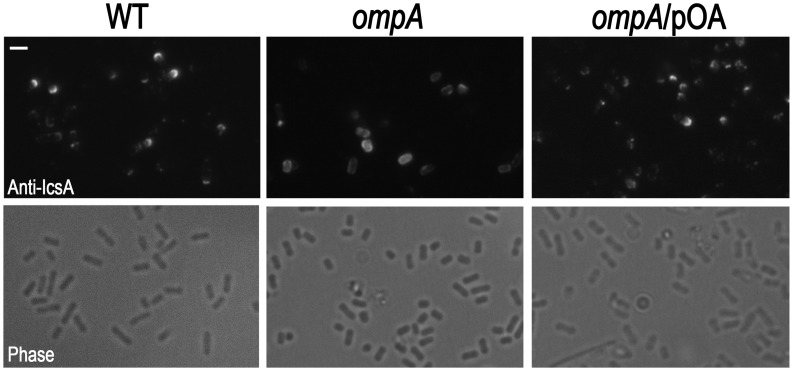
The lack of OmpA altered exposition of IcsA at *S. flexneri* lateral bacterial surface. Exponentially-growing wild-type strain (WT), the *ompA* mutant (*ompA*), and the *ompA* mutant complemented with plasmid pOA (*ompA*/pOA) were analyzed by indirect immunofluorescence using an anti-IcsA polyclonal antiserum. Representative images from at least three independent experiments are shown. Scale bar, 5 µm.

Polarly-localized IcsA is essential for ABM in *Shigella* [6], [13], [45]. To study the influence of OmpA on IcsA surface exposition at the bacterial pole, indirect immunofluorescence experiments were conducted using a polyclonal IcsA antiserum. Differently from the typical polar IcsA caps displayed by the wild-type strain, the great majority of *ompA* immunostained bacteria had detectable IcsA on the entire bacterial surface [90.0±8.7% of the *ompA* mutant displayed IcsA diffused across the entire bacterial surface; 9.5±2.2% displayed apparently normal IcsA caps and 0.5% produced polar IcsA caps smaller than wild-type strain, while 55.3±8.2% of wild-type bacteria displayed typical normal IcsA caps, 26.2±5.9% also displayed IcsA exposed at the old bacterial pole but the caps appeared smaller than typical IcsA caps, and 18.5±3.7% displayed IcsA diffused across the entire bacterial surface (*P*<0.01). Under these experimental conditions the percentage of immunostained bacteria were 26.5% for the *ompA* mutant (n = 2950), and 31.3% for wild-type strain (n = 2880) (*P*>0.5)] (Fig. 7). Noteworthy, the majority (about 62.0±7.8%) of the *ompA* mutant immunostained bacteria presented IcsA polar reinforcements (data not shown). pOA-complementation restored wild-type IcsA exposition (Fig. 7). Taken together, these results indicated that OmpA, while it is not required for the ability of *S. flexneri* to invade and multiply within host cells as well as to produce proper F-actin comet tails, it is absolutely required for protrusion formation, cell-to-cell spread and proper IcsA surface exposition.

**Figure 8:**
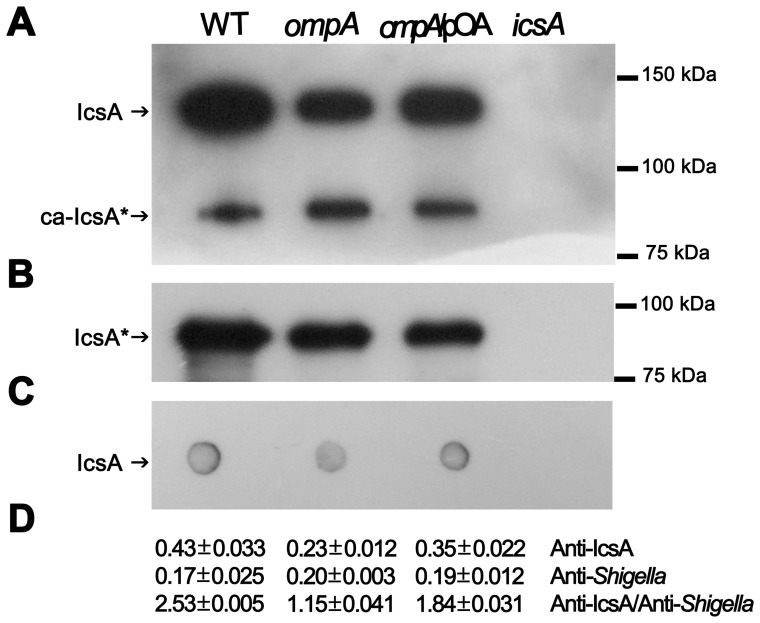
The lack of OmpA influenced IcsA expression. Western blot analysis of WCE (A) and supernatants (B) of exponentially-growing wild-type strain (WT), the *ompA* mutant strain HND92 (*ompA*), the pOA-complemented derivative strain (*ompA*/pOA) and of the *icsA* mutant strain SC560 (*icsA*). Blots were probed with an anti-IcsA antiserum. The positions of full length mature IcsA (IcsA) of IcsP-cleaved fragment (IcsA*) and of cell associated IcsA* (ca-IcsA*), are indicated by arrows at the left side. Quantitative surface immunodetection of IcsA: (C) dot blots of intact bacteria probed with anti-IcsA antibody; and (D) intact bacteria were treated with anti-IcsA and anti-*S. flexneri* antibodies. The amount of antibody bound to the bacterial surface was determined by labeling with HRP-conjugated secondary antibody and measuring the HRP enzymatic activity (OD_370_). Means and standard deviation of three independent experiments are shown. Blots are representative. Size markers (kDa) are shown at the right side.

To determine whether OmpA contributed to the presentation of IcsA on the bacterial surface, as well as to the release of IcsA* (the IcsP-cleaved 95 kDa polypeptide) into culture supernatants [26], [33], [46], WCE and culture supernatants of the *ompA* mutant, of its pOA-complemented derivative strain and of wild-type were analyzed by Western blot. Unexpectedly, when compared to wild-type, the *ompA* mutant displayed significant reduced levels of IcsA (56.6±4.8%; *P*≤0.05) and this reduced expression was due to the lack of OmpA since complementation with plasmid pOA increased these levels to 87.5±5.3% (Fig. 8A). It has been recently reported that a fraction of the IcsP-mediated cleavage products of mature IcsA remains cell associated (ca-IcsA*) so that it can be detected in WCE [33]. Densitometry analysis of WCE and of supernatant fractions revealed that wild-type, the *ompA* mutant and the pOA-complemented strain displayed similar amounts of IcsA* and of ca-IcsA* (Fig. 8A and B). These results indicated that the reduction of IcsA expression observed in the *ompA* mutant did not influence the levels of IcsP-cleaved IcsA* and ca-IcsA*.

The reduction of IcsA expression in the *ompA* mutant could be due either to reduced IcsA levels in the cell or by a reduction of the amount of IcsA detectable by antibody on the bacterial surface. To investigate these possibilities, we first investigated whether the reduction of IcsA expression was due to reduced *icsA* transcription. Real Time PCR analysis [25], conducted on cDNA preparations of wild-type and the *ompA* mutant, clearly indicated that the reduction of IcsA expression was not due to reduced transcription (data not shown). Furthermore, the amount of IcsA exposed at the surface of the *ompA* mutant and of the pOA-complemented derivative strain were determined and compared to that of wild-type by quantifying HRP enzymatic activity [32] as well as by immuno-dot blot experiments (see Materials and Methods for details). For each strain, the ratio of anti-IcsA-HRP enzymatic activity to anti-*Shigella* LPS signal was determined in order to normalize the IcsA-HRP signal to the number of bacteria. Interestingly, the HRP immunodetection assay indicated that IcsA levels on the surface of the *ompA* mutant were approximately 53.5±6.5% (ratio 0.23) compared to that of wild-type bacteria (ratio 0.43) (Fig. 8D). As expected, pOA complementation significantly restored wild-type levels (81.4±7.2; ratio 0.35). The amount of IcsA on the bacterial surface was also determined by quantitative dot-blot analysis. In agreement with the results of HRP enzymatic activity, the levels of IcsA on the bacterial surface, as measured by densitometry analysis, were found to be reduced to 51.5±5.9% of the wild-type strain, while pOA complementation increased these levels to 82.4±4.8% (Fig. 8C). Taken together, these results clearly indicated that the *ompA* mutant displayed reduced levels of antibody-accessible IcsA on the bacterial surface, that this reduction did not negatively influence the fractions of released IcsA* and of membrane-bound ca-IcsA*, and that it was indeed due to the lack of OmpA.

### Membrane Permeability of the *ompA* Mutant

OmpA has been shown to play an important role in maintaining OM integrity of *E. coli* [47], [48]. To study the effect of the lack of OmpA on membrane integrity of *S. flexneri*, we used the LIVE/DEAD BacLight kit (Invitrogen). Although this kit is commonly used to discriminate between active and dead bacteria, it is also used to differentiate between bacteria presenting intact or damaged membranes. The kit is composed of a mixture of the green- and red-fluorescent nucleic acid stains SYTO9 and propidium iodide (PI), respectively. The two fluorescent molecules differ both in their spectral characteristics and in the ability to penetrate bacterial cells. While SYTO9 enters all cells, PI enters only bacteria presenting altered membrane permeability. When used in combination, the presence of bacteria presenting defects in membrane integrity causes a reduction in the green fluorescence emission [49]. Therefore, exponentially-growing bacteria were stained with a mixture of the fluorescent dyes and the emitted fluorescence was measured and expressed in arbitrary fluorescence intensity units (see Materials and Methods for details). The results obtained indicated that while the wild-type strain displayed a green/red ratio of 20.3±1.2 units, the *ompA* mutant scored 12.3±1.0, indicating increased membrane permeability (*P*<0.01). The defect in membrane permeability was due to the lack of OmpA since complementation with pOA, increased the green/red ratio to 19.8±1.1 units, a value comparable to that of the wild-type strain. These data indicated that OmpA is required for *S. flexneri* membrane integrity.

### OmpA is not Required for Host Cytosolic Phospholipase A2 Activation

Several reports have highlighted the involvement of OmpA in pathogen-host-cell interactions [19], [50]. Recently, it has been reported that activation of host cytosolic phospholipase A2 (cPLA2α) is important for host cell invasion as well as for triggering cell cytoskeletal rearrangements [51]. In particular, using human brain microvascular endothelial cultured cells as a model to study neonatal meningitis, it has been shown that OmpA of an *E. coli* K1 strain isolated from a patient with neonatal meningitis triggers actin condensation through activation of cPLA2α-dependent pathways [50]. To examine whether the OmpA of *S. flexneri* is involved in the activation of cPLA2α, HeLa cell monolayers were infected (1 h of infection) with the wild-type and the *ompA* mutant strains. Western blot analysis of cellular lysates, using anti-p-cPLA2α (which recognizes the phosphorylated active form of cPLA2α) and anti-cPLA2α (which recognizes both the active and the inactive forms of cPLA2α) polyclonal antibodies, failed to display differences between the *ompA* mutant and the wild-type strain (Fig. S1). These results seemed to exclude that OmpA of *S. flexneri* could be involved in the activation of cPLA2α.

### Generation of an Independent *ompA* Mutant (HND99) of the *S. flexneri* Strain M90T

To confirm the results obtained with the *ompA* mutant strain HND92, we generated strain HND99 (Table 1), another non-polar *ompA* deletion mutant of the wild-type strain M90T, by using the method described by Datsenko and Wanner [52] (see Text S1 for technical details). Analysis of the virulence traits expressed by HND99 revealed that HND99 was indistinguishable to HND92. In fact, as HND92, HND99 was able to bind CR, failed to plaque on confluent HeLa cell monolayers while it was able to invade and to multiply within the cell cytoplasm at a rate similar to that of HND92 (39±8% of cells infected, and 252±18 bacteria per infected HeLa cell, 5 h post-infection) and displayed reduced protrusion formation (9.4±. 3.3% of infected cells, 1 h post-infection). Moreover, HND99 displayed IcsA exposed across the entire bacterial surface (93.2±3.0%) with the majority of immunostained bacteria presenting IcsA polar reinforcements (about 73.4±10.8%), and the levels of IcsA on the bacterial surface, as determined by immune-dot blot assay, were approximately 48.0±5.7% that of wild-type bacteria. Finally, complementation with recombinant plasmid pOA restored wild-type plaque size (0.9–1.3 mm), protrusion formation (78.5±12.3%) and proper IcsA exposition.

## Discussion

OmpA is a multifunctional major OM protein of *E. coli* and other enterobacteria [21]. Since its discovery [53], OmpA has been the subject of intense study and recently the function of OmpA as a relevant virulence factor in the pathogenesis of various infectious diseases has been recognized [19]. To our knowledge, no study concerning the relevance of OmpA in the pathogenesis of *Shigella* spp. has been reported. Moreover, it has been recently reported that OmpA of *S. flexneri* is recognized by the Toll-like receptor 2, indicating that OmpA could play a pivotal role in initiating host innate and adaptive immune responses, thus representing a potential vaccine candidate [23].

This study was aimed at examining the role of OmpA in the virulence of *S. flexneri*. To this end, an *ompA* mutant (strain HND92) of the wild-type *S. flexneri* 5a strain M90T was generated. The *ompA* mutant was able to bind Congo red, did not present growth differences with the parental strain and it was able to invade cultured epithelial cells. The percentage of HeLa cells that were invaded by the *ompA* mutant was significantly lower than wild-type, while the number of intra-cellular bacteria was higher than the number of M90T bacteria in the cytoplasm. This phenotype was highly reminiscent of *S. flexneri* mutants presenting altered cell-to-cell spread [6], [41]. Moreover, the lack of OmpA did not affect IpaABCD synthesis and secretion and the formation of apparently proper F-actin comet tails (Fig. 2 and 4), indicating that the lack of OmpA had no influence on the activation of the T3S system as well as on the IcsA-mediated activation of actin-nucleating proteins.

Indeed, the *ompA* mutant was totally impaired in cell-to-cell spread since it was unable to plaque on HeLa cell monolayers (Fig. 5). The inability to plaque was likely due to the dramatically reduced levels of protrusion formation, seen in HeLa cells infected with the *ompA* mutant. Moreover, the intra-cellular distribution of the *ompA* mutant was considerably different to that of wild-type. Bacteria did not display the classical random distribution of wild-type, while appeared to be entrapped within the cytoplasm (Fig. 3, insets). Wild-type plaque size, proper intra-cellular distribution and protrusion formation were restored by introducing plasmid pOA into the *ompA* mutant, indicating that OmpA is required for of *S. flexneri* intra- and inter-cellular spread. These findings prompted us to evaluate IcsA exposition by indirect immunofluorescence. Noteworthy, the *ompA* mutant displayed IcsA exposed across the entire bacterial surface (Fig. 7), with evident IcsA polar reinforcements noticed in the majority of immunostained bacteria. We hypothesized that the presence of these IcsA polar reinforcements may account for the ability of the *ompA* mutant to form apparently normal F-actin comet tails. The aberrant exposition of IcsA on the entire bacterial surface was not due to increased IcsA expression since we unexpectedly detected even lower levels of the 110-kDa mature IcsA in WCE of the *ompA* mutant (56.6±4.8%), compared to wild-type (Fig. 8A). Moreover, quantitative surface immuno-dot blot analysis (Fig. 8C) and immuno-assay (Fig. 8D), which measure together the levels of mature IcsA and ca-IcsA* exposed on the surface of intact bacteria, fully confirmed the reduced levels of IcsA expression. Interestingly, despite reduced mature IcsA expression, the levels of ca-IcsA* detected in WCE and of IcsA* in supernatant fractions were similar (Fig. 8A and B). Complementation with plasmid pOA restored wild-type levels of IcsA expression. Furthermore, the reduced levels of mature IcsA detected in the *ompA* mutant were not due to reduced *icsA* transcription, as determined by Real-Time PCR analysis of cDNA preparations of the *ompA* mutant and of wild-type (data not shown), indicating that the reduced IcsA expression could be due to post-trancriptional regulation. Moreover, indirect immunofluorescence experiments showed that, differently from wild-type, IcsA/ca-IcsA* were accessible to the anti-IcsA antibody across the entire bacterial surface (Fig. 7). Therefore, we concluded that the lack of OmpA likely unmasked mature IcsA/ca-IcsA* at bacterial lateral surface.

Intact LPS molecules (smooth LPS) are important for virulence of *S. flexneri* and O antigen polysaccharide chains have been shown to influence IcsA exposition on the bacterial surface and ABM within host cells [42], [43]. The inability of the *ompA* mutant to plaque and IcsA exposition across the entire bacterial surface are phenotypes reminiscent of rough LPS mutants, although rough mutants have been reported to be unable to form proper F-actin comet tails [42], [43], a characteristic not shared by the *ompA* mutant (see above). To unravel this point, the LPS structure of the *ompA* mutant was evaluated and compared to that of the parental strain either indirectly, by determining the resistance to NHS which is known to correlate with a smooth LPS, or directly by silver staining of LPS preparations. Since the *ompA* mutant presented the same level of resistance to NHS and displayed the same LPS structure of the parental strain (Fig. 6), we excluded that the inability to plaque and to present IcsA exposed across the entire bacterial surface of the *ompA* mutant was due to altered LPS biosynthesis.

The current model of the distribution of IcsA on the *S. flexneri* OM indicates that, following its secretion through the Sec translocon, IcsA is strictly delivered and inserted into the OM at the old bacterial pole by the aid of specific proteins that recognize and bind to IcsA [9], [13], [33], [54], [55]. Subsequently, membrane-bound IcsA diffuses to lateral bacterial surface by membrane fluidity, forming a gradient that decreases in concentration with distance from the old pole and IcsP is thought to be involved in the sharpening of the IcsA gradient [54], [56]. IcsA at lateral bacterial surface is likely masked by LPS molecules, probably in combination with specific OM proteins, in order to hamper its function in ABM at those sites [42], [57]. To explain the formation of typical IcsA caps in *S. flexneri* it has been hypothesized that LPS molecules of shorter length may be preferentially localized at the old cell pole, thus allowing exposition and function of IcsA only at this site [57].

Mutations that alter the synthesis or assembly of any of the OM components may cause permeability defects [58]. OmpA, an extremely abundant protein (about 100.000 molecules/cell) and a predominant antigen in enterobacterial OM, has been shown to play an important role in maintaining *E. coli* OM integrity [20], [47], [48]. By using a fluorescence approach (the BacLight kit, Invitrogen), we have shown that the lack of OmpA increased membrane permeability of *S. flexneri* [19]. Although the mechanisms for targeting and assembling proteins into the OM are largely unknown, it is possible to envisage that the lack of OmpA may have considerably altered the homeostatic control mechanism that coordinates the overall OM assembly process in *S. flexneri* [20]. In this context, it is conceivable that alteration of the OM composition due to the lack of OmpA (we have shown that OmpA is required for *S. flexneri* membrane integrity; see above), likely mislocalized newly synthesized lipids (LPS and phospholipids) as well as OM proteins, so that only a fraction of mature IcsA undergoes proper insertion into the OM. We hypothesized that the fraction of mature IcsA molecules which are not assembled into the OM might undergo protein degradation, thus accounting for the reduced IcsA expression seen in the *ompA* mutant. In line with the finding that the *ompA* mutant displayed the same LPS structure of the parental strain, it is conceivable to hypothesize OmpA as a possible LPS interactor required for the masking of mature IcsA and/or of ca-IcsA* at lateral bacterial surface.

Noteworthy, while the aberrant IcsA exposition across the bacterial surface did not influence the ability of IcsA to interact with host proteins vinculin and N-WASP to nucleate F-actin polymerization only in correspondence of the old bacterial pole (where we found IcsA polar reinforcements; see above), the lack of OmpA dramatically affected protrusion formation. Although ABM is absolutely required for protrusion formation in *Shigella* spp., the specific molecular mechanisms are poorly understood [18]. It has been recently suggested that an actin nucleation process, independent of the Arp2/3 complex, may be involved in productive protrusion formation [12]. One intriguing possibility is that OmpA of *Shigella* might also interact, directly or indirectly, with specific host proteins to trigger invasion of neighboring cells. Several host proteins like the Diaphanous formin Dia, ezrin, vinculin, and cadherins have been reported to be implicated in the process of protrusion-mediated cell-to-cell spread of *Shigella* spp. [18]. Experiments are underway to evaluate the importance of the interactions between OmpA and these host proteins in the process of protrusion formation.

Moreover, OmpA interaction with host cPLA2α has been also reported [50]. Apart from its role in liberating arachidonic acid from membrane phospholipids, cPLA2α has been shown to be important for host cell invasion as well as for triggering host cell cytoskeletal rearrangements [50], [51]. In particular, it has been demonstrated that OmpA of a neonatal meningitis causing *E. coli* K1 strain triggers actin condensation via activation of cPLA2α-dependent pathways [50]. On this basis, we compared the ability of the *ompA* mutant and of the wild-type strains to activate cPLA2α. Since no difference in cPLA2α activation was observed in extracts of HeLa cells infected with the two strains (Fig. S1), we concluded that, differently from the *E. coli* K1 strain, the OmpA of *S. flexneri* was not involved in cPLA2α activation.

Four classes of *S. flexneri* mutants defective in intercellular spread have been previously recognized [59]. Mutants unable to form protrusions as well as mutants unable to mediate their uptake into adjacent cells via unknown mechanisms were both included into the fourth class [59], [60] In this respect, the *ompA* mutant might represent a new class of *S. flexneri* mutants being able to invade and multiply within host cells, able to produce proper F-actin comet tails, presenting a smooth LPS, but exhibiting IcsA exposed across the lateral bacterial surface, impaired in plaque and protrusion formation.

The production of apparently normal F-actin comet tails displayed by the *ompA* mutant is in contrast with previous studies reporting that *S. flexneri* mutants displaying IcsA exposed across the bacterial surface presented aberrant bacterial motility [42], [43]. To explain this discrepancy we argued that the apparently normal F-actin comet tails, displayed by the *ompA* mutant, might be ineffective to generate those unidirectional movements which have been shown to be necessary for productive protrusion formation. This hypothesis is supported by the findings that not-polarly exposed IcsA drastically affects the force of actin-based motility necessary to deform the plasma membrane thus leading to protrusion formation [59], and that ca-IcsA* has been shown to be not sufficient to mediate effective ABM [33]. Therefore, we envisage that the apparently normal F-actin comet tails, displayed by the ompA mutant, are not effective to generate productive protrusion formation, thereby blocking the mutant within the cytoplasm.

The observed phenotype of the *ompA* mutant was reminiscent of that displayed by a *mlaA*/*vacJ S. flexneri* mutant (*mlaA*/*vacJ* encodes a lipoprotein exposed on the bacterial surface) [41]. It has been reported that the *mlaA*/*vacJ* mutation, generated into the *S. flexneri* 2a strain YSH600T, did not affect the ability of S. *flexneri* to invade and to multiply within infected MK2 cells and to form protrusions at a rate similar if not identical to that of the wild-type strain, while the *mlaA*/*vacJ* mutant was unable to plaque and presented increased membrane permeability. The authors reported that the defect in plaque formation was due to the inability of the mutant to move from protrusions into the cytoplasm of adjacent cells but they did not evaluate the exposition of IcsA on the bacterial surface [41]. This latter point would have been crucial to assess similarity between these two mutants. Anyway, since the authors reported that the *mlaA*/*vacJ* mutant displayed wild-type levels of bacterium-containing membranous protrusions, while the *ompA* mutation dramatically affected protrusion formation, this difference appeared to rule out the possibility that the lack of OmpA may exert a pleiotropic effect on the expression of the *mlaA*/*vacJ* gene.

Nevertheless, the results presented in this study have received further support by the analysis of HND99, an independent *ompA* mutant of the wild-type *S. flexneri* strain M90T (Table 1; Text S1). HND99 was found to be indistinguishable to the *ompA* mutant strain HND92. In fact, HND99 was able to invade and to multiply within infected HeLa cells, displayed reduced protrusion formation, failed to plaque and presented IcsA exposed across the entire bacterial surface, with the majority of immunostained bacteria displaying IcsA polar reinforcements. Moreover, as HND92, the introduction of recombinant plasmid pOA into HND99, restored wild-type levels of the considered virulence phenotypes.

Taken together, we concluded that OmpA is required and sufficient for the expression of relevant virulence phenotypes as formation of typical IcsA caps, plaquing ability and protrusion formation and thus for intra-and inter-cellular *S. flexneri* motility (Fig. 3, 5 and 7). Since the membrane architecture of the spatial arrangement of OmpA depends on the presence and on the interaction with a variety of other molecules [20], we could not exclude that OmpA might play only an indirect role in the expression of these virulence phenotypes. Experiments are in progress to unravel this point.

In conclusion, in this study we present substantial experimental evidence demonstrating that OmpA plays an active role in relevant *S. flexneri* virulence traits, like IcsA exposition, cell-to-cell-spread and protrusion formation. These findings complement the recently discovered passive role of OmpA in the induction of the host immune responses [23], strenghthening its importance also as a promising vaccine candidate.

## Supporting Information

Text S1 Construction of HND99, a non-polar *ompA* deletion mutant of the wild-type *S. flexneri* strain M90T.
(DOC)

Figure S1 **OmpA of** ***S. flexneri*** **is not involved in cPLA2α activation.** HeLa cell monolayers were infected with the wild-type (WT) and the *ompA* mutant (*ompA*) strains, at a MOI of 20. At 1 h post-infection, cells were lysed and immunoblotted using anti-p-cPLA2α and anti-cPLA2α rabbit polyclonal antibodies. A lysate of uninfected HeLa cells (Ctrl) were included as control.
(TIF)
