# Supplementary material for: Outer Membrane Protein A (OmpA): A New Player in Shigella flexneri Protrusion Formation and Inter-Cellular Spreading
Source: PLoS One. 2012 Nov 14;7(11):e49625. doi: 10.1371/journal.pone.0049625 (PMC3498225; doi:10.1371/journal.pone.0049625)
Supplement: Text S1 — Construction of HND99, a non-polar ompA deletion mutant of the wild-type S. flexneri strain M90T. (DOC) [file pone.0049625.s001.doc]

**Text S1.** Construction of HND99, a non-polar *ompA* deletion mutant of the wild-type *S. flexneri* strain M90T. A non-polar deletion of *ompA* was introduced into *S. flexneri* strain M90T also by allelic exchange using the PCR-based method described by Datsenko and Wanner [52], using the specific primer pair ompADFW and ompADRV (Table 1). Primers were used to amplify the kanamycin-resistance (Km) cassette from pKD4 with sequences at the 5’ and 3’ ends identical to sequences 40 bp and 39 bp internal to *ompA*. After transformation, bacteria were plated on CR plates supplemented with kanamycin for selection of recombinants. The Km resistance cassette was removed by transforming with pCP20 and incubating selected mutants at 42°C [52]. One of these mutants, strain HND99, was used for further studies. Correct introduction of the expected *ompA* deletion was verified by PCR (using the primer pair ompA2FW and ompA2RV; Table 1) and sequencing.
