# Supplementary figures and images for: Outer Membrane Protein A (OmpA): A New Player in Shigella flexneri Protrusion Formation and Inter-Cellular Spreading
Source: PLoS One. 2012 Nov 14;7(11):e49625. doi: 10.1371/journal.pone.0049625 (PMC3498225; doi:10.1371/journal.pone.0049625)

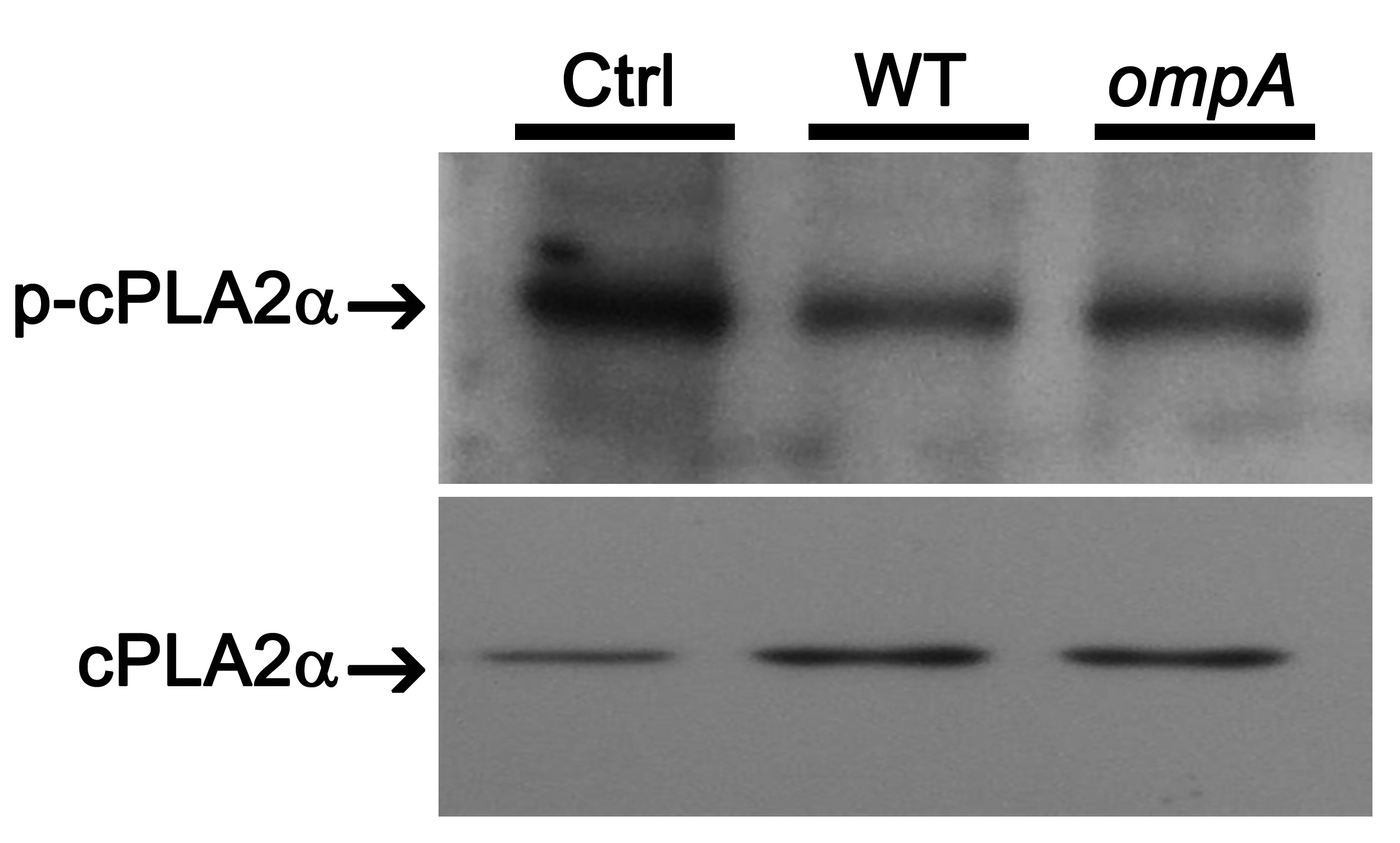

Supplement: Figure S1 — OmpA of S. flexneri is not involved in cPLA2α activation. HeLa cell monolayers were infected with the wild-type (WT) and the ompA mutant (ompA) strains, at a MOI of 20. At 1 h post-infection, cells were lysed and immunoblotted using anti-p-cPLA2α and anti-cPLA2α rabbit polyclonal antibodies. A lysate of uninfected HeLa cells (Ctrl) were included as control. (TIF) [file pone.0049625.s002.tif]
